# Protocol for the ELISPOT-TC Trial: A Randomized Controlled Study Evaluating CMV-Specific Cellular Immune Monitoring in Heart Transplant Recipients

**DOI:** 10.3389/ti.2025.15565

**Published:** 2025-11-27

**Authors:** Elena García-Romero, Carles Díez-López, Delphine Kervella, Víctor Donoso, María Dolores García-Cosío, Carlos Ortiz-Bautista, Francisco José Hernández-Pérez, David Couto-Mallón, Francisco González-Vílchez, Luis De la Fuente-Galán, Laura López-López, Antonio Grande-Trillo, Miríam Gómez-Molina, Pablo Catalá, Lorena Herrador, Laia Rosenfeld, Silvia Ibáñez, Laura Donadeu, Nuria Sabé, Josep Comín-Colet, Oriol Bestard, José González-Costello

**Affiliations:** 1 Cardiology Department, Bellvitge University Hospital, Barcelona, Spain; 2 Bio Heart Cardiovascular Diseases Research Group, Bellvitge Biomedical Research Institute (IDIBELL), Barcelona, Spain; 3 Ciber Cardiovascular group (CIBER-CV), Instituto Salud Carlos III, Madrid, Spain; 4 Universitat de Barcelona, Barcelona, Spain; 5 Experimental Nephrology Laboratory, Vall d’Hebron Research Institute, (VHIR), Barcelona, Spain; 6 Cardiology Department, Hospital Universitari i Politecnic La Fe, Valencia, Spain; 7 Cardiology Department, Hospital Universitario 12 de Octubre, Madrid, Spain; 8 Cardiology Department, Hospital General Universitario Gregorio Maranon, Madrid, Spain; 9 Cardiology Department, Hospital Universitario Puerta de Hierro Majadahonda, Majadahonda, Spain; 10 Cardiology Department, Complexo Hospitalario Universitario A Coruna, A Coruña, Spain; 11 Cardiology Department, Hospital Universitario Marques de Valdecilla, Santander, Spain; 12 Cardiology Department, Hospital Clinico Universitario de Valladolid, Valladolid, Spain; 13 Cardiology Department, Hospital de la Santa Creu i Sant Pau, Barcelona, Spain; 14 Cardiology Department, Hospital Universitario Virgen del Rocio, Seville, Spain; 15 Cardiology Department, Hospital Clínico Universitario Virgen de la Arrixaca, Murcia, Spain; 16 Infectious Diseases Department, Bellvitge University Hospital, Barcelona, Spain; 17 Nephrology Department, Hospital Universitari Vall d'Hebron, Barcelona, Spain

**Keywords:** cytomegalovirus (CMV), cellular immune response, heart transplantation, ELISpot, enzyme-linked immunosorbent spot

Dear Editors,

Human cytomegalovirus (CMV) is the most frequent opportunistic infection in the early months after heart transplantation with reported DNAemia in up to 40%–50% of high risk recipients and CMV disease in 10%–15% within the first year post-transplantation. CMV continues to exert a major negative impact, contributing to acute rejection, opportunistic infections, coronary allograft vasculopathy, graft dysfunction and increased healthcare costs [1].

Post-transplant risk stratification is based on donor–recipient serostatus (D+/R–as high risk, R+ as intermediate, and D–/R–as low); paradoxically CMV transmission and infection may still occur even in settings where immunity or prophylaxis should confer protection, reflecting organ-specific variability in transmission risk and indicating that serology alone does not reliably reflect protective immunity, thereby highlighting the need for additional immune biomarkers [2].

Memory/effector T cells help control CMV through responses to IE-1 and pp65 antigens and the IFN-γ ELISpot assay enables the detection of CMV-reactive T cells and prediction of infection risk [3]. CMV-specific cellular immunity (CMI) can identify higher-risk kidney transplant recipients who are more likely to develop CMV infection, while preserved CMV-CMI responses confer protection against post-transplant replication and disease [4, 5]. Recent multicenter trials have further supported the role of immunoguided prophylaxis in solid-organ transplantation. In kidney and liver transplant recipients, immune monitoring based on CMV-specific T-cell responses safely reduced antiviral exposure without increasing infection rates [6], whereas in lung transplantation, CMV-CMI–guided prophylaxis proved non-inferior to standard strategies while minimizing antiviral toxicity and adverse events [7].

However, most studies assessing CMV-specific cellular immunity have been conducted in kidney transplantation, and their findings may not be fully extrapolable to heart transplant recipients, who face higher perioperative morbidity and mortality [5].

Current Spanish and international guidelines for heart transplantation provide only low-level evidence, and CMV-CMI monitoring is still recommended based solely on expert opinion, particularly in seropositive (R+) recipients [8, 9].

Preventive strategies currently rely on universal prophylaxis with valgancicloviror PCR-guided preemptive therapy. While effective, both have limitations such as toxicity, late-onset infections, and viral resistance [8, 10].

The ELISPOT-TC trial will address this gap by evaluating whether prophylaxis guided by CMV-specific CMI, assessed by IFN-γ ELISpot (T-SPOT.CMV), is non-inferior to universal valganciclovir prophylaxis in seropositive heart transplant recipients.

It will be a phase IV, multicenter, open-label, randomized (2:1), non-inferiority clinical trial conducted across 11 Spanish transplant centers. A total of 188 adult CMV-seropositive recipients will be enrolled (125 experimental, 63 control) as shown in Figure 1.

**FIGURE 1:**
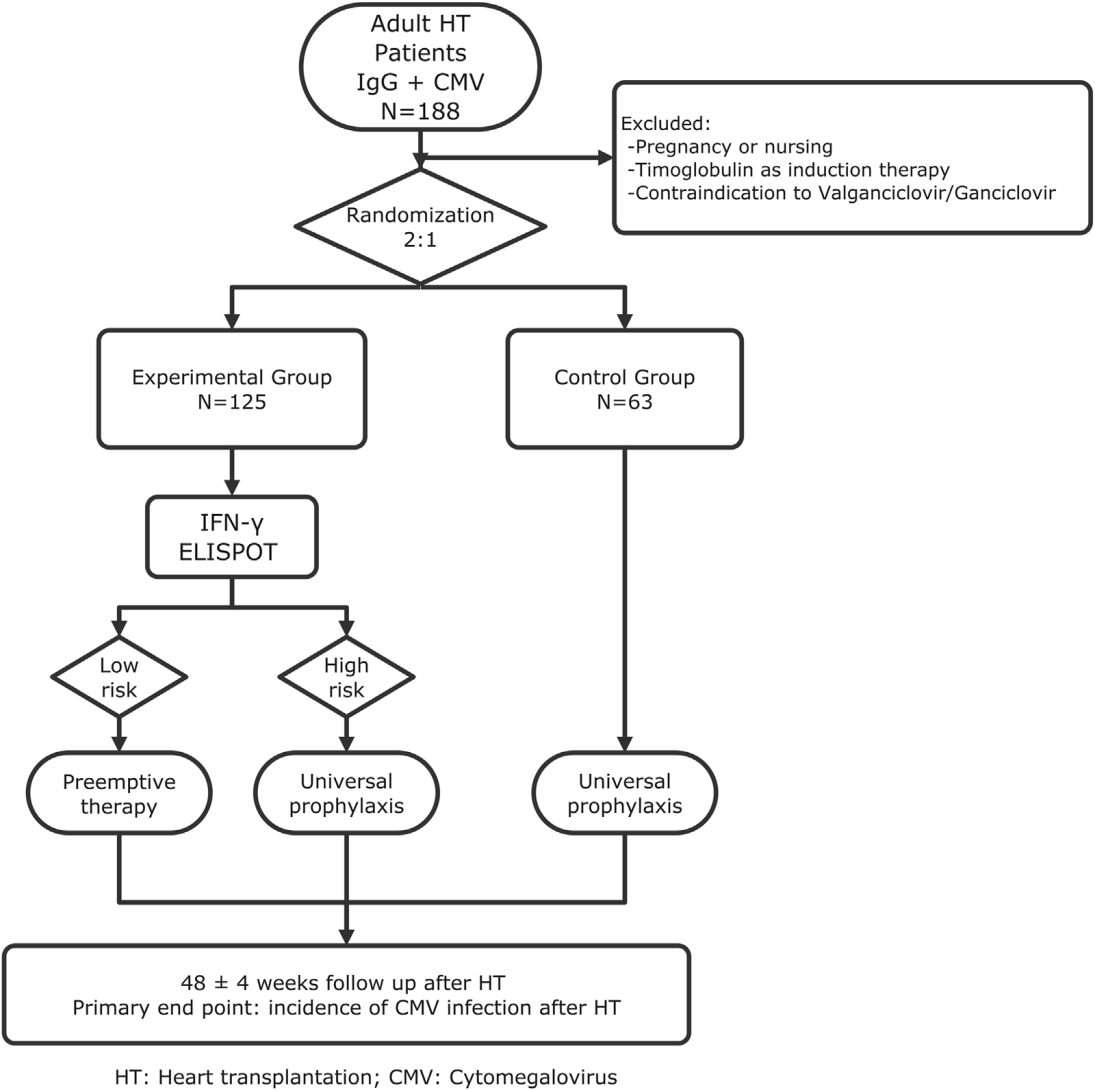
Flowchart of the trial.

Patients will be randomly assigned by a centralized computer system to either:

- Group 1: CMI-guided prevention based on T-SPOT. CMV results, or
- Group 2: Standard universal valganciclovir prophylaxis for 3 months.

Randomized will be centralized and stratified by center. The assigned preventive strategy will not be masked to investigators or participants.

Eligible participants will be adult (≥18 years) CMV-seropositive recipients able to provide informed consent. All patients will undergo T-SPOT. CMV testing at day 10 and at month 3 post-transplant,Viral load monitoring by quantitative nucleic acid testing (QNAT) will be performed throughout follow-up according to a standardized schedule.

In the experimental arm, T-SPOT. CMV testing at day 10 post-transplant will classify patients as high or low risk, guiding either initiation of antiviral prophylaxis in those without CMV-specific cellular immunity (high-risk) or a pre-emptive strategy in those with preserved immunity (low-risk).

Patients will be followed for 48 weeks with scheduled visits to monitor outcomes.

The primary endpoint will be the cumulative incidence of CMV infection at 1 year. Secondary endpoints will include the classification of CMV infection into clinically significant and no-clinically significant categories (according to standardized, consensus-based viral load thresholds uniformly applied across centers and the presence of CMV-related symptoms or disease) as well as CMV disease, graft rejection, opportunistic infections, hematological adverse events, mortality, and cost-effectiveness.

Analysis is planned both per-protocol and by intention-to-treat. Comparisons between groups will use standard tests for continuous and categorical variables, Cox proportional hazards models for time-to-event analyses, and Kaplan-Meier survival curves. Non-inferiority will be assessed with a 10% margin. The trial will comply with the Declaration of Helsinki and Spanish legislation and has received approval from the national regulatory authority and local ethics committees. It has been registered at ClinicalTrials.gov (NCT04278547).

This study will represent the first randomized multicenter trial in heart transplantation to use a validated and commercially available IFN-γ ELISpot assay (T-SPOT.CMV) for guiding CMV prevention. As a randomized clinical trial, the study design will ensure strong internal validity and methodological robustness, while stratification by center will enhance the generalizability of the results across diverse clinical settings. By prospectively integrating immunological monitoring into a clinical decision algorithm, this trial takes an essential step toward individualized prevention.

The trial will not only examines efficacy and safety but will also address the economic impact, including both direct antiviral costs and indirect healthcare-related costs.

Universal valganciclovir prophylaxis is effective but costly and associated with frequent adverse events such as leukopenia and neutropenia. A CMI-guided approach has the potential to reduce unnecessary drug exposure, minimize toxicity, and lower costs while maintaining protection. This aligns with the broader movement toward precision medicine in transplantation [8, 9].

Certain limitations should be acknowledged: variability among participating laboratories may influence CMV DNAemia quantification despite standardized procedures; the exact proportion of patients who will ultimately receive valganciclovir prophylaxis versus preemptive therapy cannot be determined in advance; and the 48-week follow-up period will be insufficient to fully assess indirect CMV effects such as cardiac allograft vasculopathy, which has traditionally been one of the main arguments supporting universal prophylaxis.

Despite these challenges, the ELISPOT-TC trial could support a paradigm shift in CMV prevention. Instead of exposing all patients to antiviral prophylaxis, immune monitoring could allow for targeted use of therapy, reducing adverse events and improving cost-effectiveness. The ELISPOT-TC trial thus represents a landmark step toward personalized prevention strategies in transplantation [9].

Detailed methods, inclusion criteria, statistical analyses, and full CONSORT checklist will be provided in the Supplementary Material.

Sincerely,

## Data Availability

The raw data supporting the conclusions of this article will be made available by the authors, without undue reservation.
