# Supplementary material for: Protocol for the ELISPOT-TC Trial: A Randomized Controlled Study Evaluating CMV-Specific Cellular Immune Monitoring in Heart Transplant Recipients
Source: Transpl Int. 2025 Nov 27;38:15565. doi: 10.3389/ti.2025.15565 (PMC12695717; doi:10.3389/ti.2025.15565)
Supplement: Supplementary file 1 [file Supplementaryfile1.docx]

Supplemental Digital Content

1. Study design and Setting:

The ELISPOT-TC trial will be a phase IV, multicenter, open-label, randomized (2:1) non-inferiority clinical trial conducted across 11 Spanish heart-transplant centers. The trial will evaluate a CMV-specific cellular immunity (CMI)-guided prevention strategy using the IFN-γ ELISpot (T-SPOT.CMV) assay versus standard universal valganciclovir prophylaxis for 3 months. Patients will be followed for 48 weeks after transplantation.

1. Study Objectives and Endpoints:

The primary objective of the ELISPOT-TC trial will be to estimate and compare the cumulative incidence of cytomegalovirus (CMV) infection during the first year after heart transplantation among CMV-seropositive (IgG-positive) recipients assigned to either a CMI-guided prevention strategy or standard universal valganciclovir prophylaxis.

The primary endpoint will be the cumulative incidence of CMV infection at one year post-transplant. CMV infection will be categorized as clinically significant or non-significant according to predefined standardized thresholds agreed upon across centers:

Clinically significant CMV infection will be defined as CMV DNAemia ≥ 1000 IU/mL or ≥ 500 copies/mL in plasma, or ≥ 4500 IU/mL or ≥ 3000 copies/mL in whole blood, or any detectable viral replication associated with CMV syndrome or tissue-invasive disease.

Non-significant CMV infection will include episodes of CMV DNAemia below these thresholds without clinical manifestations.

Secondary endpoints will include the incidence of CMV disease, acute graft rejection, opportunistic infections, hematologic adverse events, all-cause mortality, and cost-effectiveness of both preventive strategies.

1. Participants:

Eligible participants will be adult (≥18 years) CMV-seropositive (IgG-positive) heart transplant recipients of either sex. Written informed consent will be obtained before inclusion. Exclusion criteria will include pregnancy or lactation, administration of thymoglobulin as induction therapy, or any contraindication to receive ganciclovir or valganciclovir. Fertile women will be required to use effective contraception for at least 30 days after treatment, and men for 90 days unless the partner is not at risk of pregnancy.

1. Randomization and Allocation Concealment:

Participants will be randomized using a centralized computer-generated system in a 2:1 ratio (experimental:control), stratified by center. The allocation sequence will be generated electronically by the coordinating center and implemented via a secure web-based platform to ensure concealment. Due to the nature of the interventions, masking of investigators and participants will not be feasible.

1. Interventions:

*Assessment of CMV-specific Cell Mediated Immunity:*

The commercially available IFN-γ ELISpot assay T-SPOT®.CMV (Oxford Immunotec, ltd., Oxford, UK) will be used to assess the CMI against 2 major immunogenic CMV antigens (IE-1 and pp65) using overlapping peptide

pools covering the whole antigen length and has been conducted following manufacturer guidelines. Briefly, 2.5x10^5^ PBMC (in 100µl) will be stimulated with CMV antigens for 20 hours in duplicate wells. IFN-ɣ spots will be detected after using biotinylated anti-human IFN-ɣ antibody plus the addition of alkaline phosphatase conjugate substrate. IFN-ɣ spots obtained will be counted with an ELISPOT reader (AID® Gmbh, ELISPOT Reader HR fourth generation). In each test, AIM-V medium alone and Phytohemagluttinin (PHA) will be used as negative and positive controls, respectively. Total CMV-specific IFN-ɣ spots will be provided after subtracting the number of IFN-ɣ spots observed in the respective negative controls.

The cut-offs that will be used for decision-making are as follows:

**Low CMV risk:** >40 IFN-γ spots/2.5 x10^5^ for IE-1 antigen and >170 IFN-γ spots/2.5 x10^5^ for pp65 antigen

**High risk:** <40 IFN-γ spots/2.5 x10^5^ for IE-1 antigen and <170 IFN-γ spots/2.5 x10^5^ for pp65 antigen or IE-1<40 and pp65>170 or IE-1>40 and pp65<170

**Unreactive:** <5 IFN-γ spots/2.5 x10^5^ for IE-1 antigen and <5 IFN-γ spots/2,5 x10^5^ for pp65 antigen and positive control with 5-50 IFN-γ spots/2.5 x10^5^ for both antigens.

Samples yielding an undetermined T-SPOT.CMV result will be repeated. Samples classified as unreactive will be considered high-risk.

Only 10-days post-transplant IFN-γ T-SPOT.CMV assays will be performed using fresh peripheral blood samples in group 1 in order to stratify patients, whereas pre-transplant, 10 days post-transplant from group 2 and end of treatment (three months) immune assays will be performed using frozen samples, isolated and cryopreserved in liquid nitrogen for its subsequent evaluation at the end of the trial.

Samples will arrive at the laboratory within 24 hours after extraction and will be processed within 32 hours with the T-cell Extend® reagent. This reagent is an antibody complex that is added to blood samples in the laboratory immediately before running the T-SPOT.TB assay and allows blood samples to be processed up to 32 hours after venepuncture without affecting the accuracy of the test.

*CMV Serology and Microbiological studies:*

Pre-transplant CMV serological status of all donor and recipients will be determined using a Human Anti-CMV IgG Enzyme-Linked ImmunoSorbent Assay (ELISA) kit in each center.

Surveillance of CMV DNAemia will be performed with real-time polymerase

chain reaction kit in plasma or whole blood in each center laboratory. Results will be despicted as IU/mL or copies/mL. CMV DNAemia will be considered positive when detectable above 1000 IU / mL or 500 copies / mL in plasma or above 4500 IU / mL or 3000 copies / mL in whole blood.

*CMV Preventive strategies:*

Patients randomized at ELISPOT group (group 1) will be stratified based on the result of the IFN-γ T-SPOT.CMV assays into high risk of developing CMV infection or low risk of developing CMV infection.

Those patients classified as high risk for developing CMV infection (group 1 a) due to a high risk, undetermined or unreactive result will receive **universal prophylaxis** with valganciclovir 900 mg / 24h for 3 months (and adjusted to the glomerular filtrate rate of the patient). In case prophylaxis can’t be done orally, intravenous ganciclovir will be used at 5-10 mg / kg / day for 1-3 months.

In these patients, treatment will begin once the result is obtained, between days 10 and 15 post-transplant, taking into account that there is a window of 10 +/-2 days for extraction of the sample, which will be received in 24-36 hours in the central laboratory, obtaining the result 48 hours after the extraction. If the sample is not valid, the extraction and determination can be repeated, as long as it allows prophylaxis to begin at the latest on day 15 post-transplant, so as not to increase the risk of CMV infection.

Patients randomized to control group (group 2) will receive **universal prophylaxis** with valganciclovir 900 mg / 24h for 3 months (and adjusted to the glomerular filtrate rate of the patient). In case prophylaxis can’t be done orally, intravenous ganciclovir will be used at 5-10 mg / kg / day for 1-3 months. Prophylaxis can be started after sample extraction (day 10 +/- 2 days post-HT) and no later than day 15 post-HT, therefore maintaining the same window period to start prophylaxis in both groups (group 1a and control group).

*Surveillance of CMV:*

After stopping prophylaxis in both groups, patients will be followed preemptively with CMV Quantitative Nucleic Acid Testing (QNAT) every month until month 6 and at month 12 after transplantation.

Patients randomized to the intervention group who present a low-risk T-SPOT.CMV result (group 1b) will follow a **preemptive therapy**, consisting in weekly QNAT during the first month, every two weeks until month 3, monthly until month 6 and at month 12 after transplantation. Antiviral treatment will be initiated in those patients with a DNAemia above ≥1000 IU / mL or ≥500 copies / mL in plasma (or ≥4500 IU / mL or ≥3000 copies / mL in whole blood) without associated symptoms or the presence of any level of replication with associated viral syndrome or CMV disease, ultimately being at the discretion of the attending physician. Patients will be treated with valganciclovir 900 mg / 12 h or ganciclovir 5 mg / kg every 12 hours (adjusted according to renal function and possible adverse events, at the discretion of the attending physician), a minimum of 14 days in case of asymptomatic DNAemia or a minimum of 21 days in case of CMV viral syndrome o CMV disease. If patients cannot receive valganciclovir or ganciclovir, other antivirals are allowed. To stop treatment, 2 negative viral load results must be obtained in 2 consecutive determinations obtained at least 1 week apart.

QNAT will be performed on all patients at the time of sample extraction for IFN-γ T-SPOT.CMV assay (10 +/-2 days post-HT and three months after trasplantation).

If DNAemia is detected in either of the two groups that will need prophylaxis, before starting it, they will continue to be eligible for prophylaxis as long as they do not exceed the following cut-off points that we have considered to be clinically significant (as explained below).

QNAT will also be performed on all patients at discharge, at 3 months (12 ± 4 weeks), 4 months (16 ± 4 weeks), 5 months (20 ± 4 weeks), 6 months (24 ± 4 weeks) and one year (48 ± 4 weeks) post-HT in the local laboratories of each center.

In patients receiving prophylaxis (group 1 a and control group) QNAT will be also performed at 15 days (± 3 days) after the end of treatment.

Although no clinical guidance has been provided on the treatment of late disease, the treatment received will be recorded.

1. Sample Size Calculation and Statistical Analysis:

Sample size will be dtermined assuming a 40% cumulative incidence of CMV infection at one year, a one-sided type I error of 2.5%, 80% statistical power, and a 10% non-inferiority margin. A total of 188 patients (125 experimental and 63 control) will be required, accounting for an anticipated 10% dropout rate. Analyses will be conducted on both an intention-to-treat and per-protocol basis. Comparisons between groups will use standard parametric or nonparametric tests as appropriate. Time-to-event analyses will be performed using Cox proportional hazards models and Kaplan–Meier survival curves. Cost-effectiveness analyses will include both direct and indirect healthcare costs. All statistical tests will be two-sided, and p-values <0.05 will be considered significant.

1. Ethical Approval:

The trial will comply with the Declaration of Helsinki and Spanish legislation. Approval has been obtained from the national regulatory authority and all local ethics committees at participating centers. All patients will provide informed consent before any study procedure. The study has been registered at ClinicalTrials.gov (NCT04278547).

1. Funding:

This work will be supported by the Instituto de Salud Carlos III (Grant number PI19/01610), co-founded by the Spanish Cardiology Society (Grant number 19PSJ017) and by the Spanish Platform for Clinical Research and Clinical Trials, SCReN (Spanish Clinical Research Network), financed by the ISCIII-General Subdirectorate of Evaluation and Promotion of Research, through project PT17/0017/0010 and PT20/00008 integrated into the I+D+I 2013-2016 State Plan and co-financed by the European Regional Development Fund (FEDER). We thank *CERCA Programme / Generalitat de Catalunya* for institutional support.
